# Focal therapy in prostate cancer: the current situation

**DOI:** 10.3332/ecancer.2014.435

**Published:** 2014-06-10

**Authors:** FX Jácome-Pita, R Sánchez-Salas, E Barret, N Amaruch, C Gonzalez-Enguita, X Cathelineau

**Affiliations:** 1 Urology Department, Institut Montsouris, Paris 75014, France; 2 Urology Department, Fundación Jiménez Díaz, Madrid 28040, Spain

**Keywords:** localised prostate cancer, focal therapy

## Abstract

Prostate cancer is one of the most significant pathologies in the field of urology. The adoption of screening strategies and improvements in biopsies have resulted in an increase in early-stage tumour detection.

Radical global therapies provide very good oncological results in localised prostate cancer. However, excess treatment in low- and, in some cases, intermediate-risk groups affects the quality of life of these patients.

In the case of localised prostate cancer, focal therapies offer a minimally invasive option with good results with respect to established treatments. Although this is currently not a standard treatment, it represents the therapeutic approach with the greatest potential.

This literature review has the following objectives: to define selection criteria for patients who are candidates for focal therapy, to assess the current situation and results of the different therapeutic options, and to define procedures in cases of recurrence and for follow-ups.

We concluded that focal therapy is a viable therapeutic alternative for localised prostate cancer, specifically cryosurgery and high-intensity targeted ultrasound, which have acceptable oncologic results and a lower comorbidity compared with global treatments. Studies with a high level of scientific evidence are still needed to validate these results.

**Acquisition of evidence:**

A search was carried out on the Medline (PubMed), EMBASE, Web of Science and Cochrane databases of all papers published before 31 July 2013. We included clinical studies and literature reviews that evaluated primary focal therapy for prostate cancer confirmed by biopsy and excluded focal rescue therapy studies. The keywords used were focal therapy and prostate cancer.

Initially, we found 42 articles; 15 studies were excluded because they did not meet the minimum criteria for inclusion. A total of 1350 cases were treated throughout 27 studies.

## Introduction

Prostate cancer (CaP) is one of the most significant pathologies in the field of urology. In Europe, it is the most common solid neoplasm, with an incidence of 214 cases per 1000 inhabitants [1]. In addition, CaP is currently the second leading cause of cancer-related deaths in men [2].

During the past two decades, an increase in the early diagnosis of CaP has been observed due to the adoption of formal and informal screening strategies using a prostate-specific antigen (PSA) and multi-cylinder prostate biopsies. This has allowed the identification of smaller tumours at an early stage, thereby increasing the number of cases in the low-risk group [2].

Currently, global therapies, such as radical prostatectomy, external radiotherapy, and brachytherapy, provide excellent results for cancer control in low-risk patients [4–6]. However, these therapies have two disadvantages: they entail a high incidence of adverse reactions that deteriorate the quality of life [7], and treatment could be excessive considering that many small, localised, and well-differentiated CaPs will not progress [10].

Large-scale studies, such as The Prostate, Lung, Colorectal and Ovarian Cancer Screening Trial (PLCO), SPCG-4, PIVOT, and ERSPC [8–11], have been designed to answer some of the most important questions about screening, overdiagnosis, and management. The PLCO study, which assessed the effectiveness of CaP screening, concluded that specific mortality is very low, and there are no significant differences in both groups. The SPCG-4 study compared a wait-and-see approach with radical prostatectomy in T1-T2 clinical stage cancer, which revealed a significant reduction in specific and global mortality, metastatic risk, and local progression risk with radical prostatectomy. The PIVOT study compared radical prostatectomy versus a wait-and-see approach, and found no significant differences in global and specific mortality in both groups. Finally, the ERSPC study has shown that treatment reduces mortality up to 20%; however, overdiagnosis and treatment produce adverse effects that impair quality of life, especially in the low-risk group.

A wait-and-see approach with active monitoring is presented as a viable option [3]. However, this produces significant psychological stress in the patient, and it is difficult for the doctor to propose a therapeutic option in selected patients.

This is where focal therapy seems to offer a minimally invasive therapeutic option with acceptable oncological results and probably less associated morbidity with respect to radical treatments [25–30, 32–34, 46, 47]. Although focal therapy is not currently the standard treatment for men with localised CaP, it represents the therapeutic approach with the most significant potential [24].

Currently, one of the disadvantages of focal therapy is the usual multi-focality of CaP demonstrated in several studies [12–14]. New work has shown that only the volume of the largest lesion is predictive of disease progression [13, 15, 16]. 

Therefore, the criteria for patient selection need to be clearly defined; the location, relevance, and characterisation of the cancer need to be pinpointed; and long-term focal therapy treatment results need to be evaluated.

## Objectives

The objectives of focal treatment are the following [25]:

– Optimise risk stratification at the time of diagnosis.
– Eradicate abnormalities identified for effective cancer follow-up.
– Preserve areas not affected by cancer.
– Minimise impact on quality of life.
– Provide retreatment with focal or global therapy, if necessary.

It is necessary to improve CaP diagnostic tools to optimise patient stratification in searching for the best candidates for focal therapy. Contemporary prostate imaging and biopsy methods have improved the characterisation of pre-treatment CaP in terms of location, extent, and biological potential [2].

Some imaging modalities show potential for intra-prostate staging, focal therapy guiding, and CaP follow-up. These include the use of ultrasound (Doppler, contrast, characterisation algorithms) and multi-parameter magnetic resonance imaging (MRI). However, up to now there is no standardised technique that offers better results in terms of sensitivity, specificity, predictive value, and reproducibility [17].

Prostate biopsies provide predictive pathology information such as extracapsular extension, tumour volume, and post-treatment progression. Traditionally, the standard treatment is the systematic sextant biopsy described by Hodge. However, this method has proved to be inadequate in identifying significant tumours (>0.5 cc) [18]. Even the transrectal saturation biopsy that greatly increases the number of samples did not detect significant lesions in 31% of patients [19]. Three-dimensional template-guided transperineal mapping biopsy is the method of choice for diagnosis of CaP patients who are candidates for focal therapy, because it provides for greater accuracy in preliminary studies [20]. The biggest drawback of this method is its invasiveness, which is associated with temporary complications [21].

**Figure 1.**
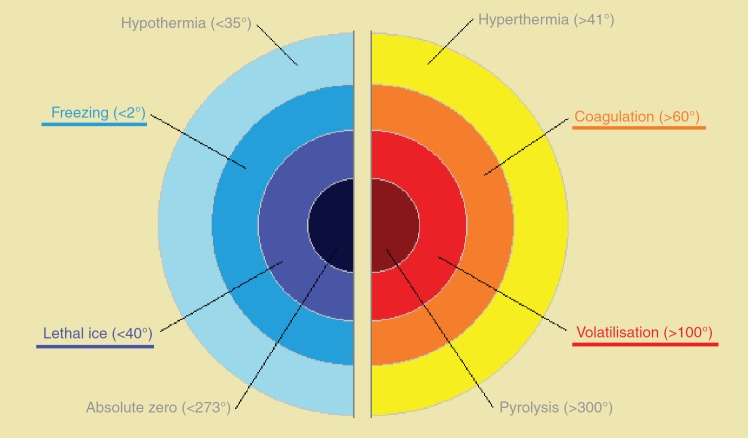
Classification of damages induced by energy.

Once the focal therapy candidate is identified, it is necessary to eradicate the affected zone for effective cancer control. There are many different focal therapy modalities. Traditionally, thermal energy is used (Figure 1) with cold or heat, although new modalities are currently being explored [37, 48, 52]. So far, cryotherapy and high-intensity focused ultrasound (HIFU) are the most frequently used methods. Currently, cryotherapy is considered an alternative therapy in the EAU and the American Urological Association (AUA) clinical guidelines [24]; HIFU is still considered an experimental therapy. In addition, several well-developed techniques such as photodynamic therapy (PDT), focal laser ablation (FLA), targeted radiation therapy (external and brachytherapy), and other techniques being developed (radiofrequency, electroporation, nanoparticles, etc.) represent therapeutic approaches.

There are currently no standardised rules concerning which focal therapy is best for each type of patient. However, we can evaluate the limitations of each method before its implementation (Table 1).

One of the most important advantages of targeted therapies is to preserve areas not affected by CaP. This, in turn, contributes to minimising the impact on the patient’s quality of life. Numerous pilot studies show these findings [25–30, 32–34, 46, 47].

Finally, it is necessary to establish optimal patient follow-up and procedures to follow in cases of recurrence. The most logical option would be retreatment with the same approach, and the same energy. It is necessary to take into account the lesion’s new characteristics, the PSA levels and kinetics, and the limitations of each focal therapy. In addition, we must remember that it is always possible to use a new technique.

## Current situation

### Prostrate cryosurgery

Cryosurgery uses freezing techniques to cause cell death by denaturing protein by dehydration; rupturing cell membranes by crystallisation; microvascular stagnation by stasis and microthrombosis with consequent tissue ischaemia and apoptosis [22].

Freezing of the prostate is carried out by placing cryoneedles transrectally using ultrasound to guide placement (TRUS). Two cycles of freezing and thawing are used, which result in temperatures of −40 °C. This technique relies on pressurised gas, using argon in the freezing phase and helium in the thawing phase.

Placing thermosensors at the external anal sphincter, prostate apex, and neurovascular bundle and using a urethral heater contribute to reduction in complications.

Two parameters are related to the degree of cellular destruction: the speed of freezing and the lowest temperature attained.

The TRUS allows monitoring during the procedure through the display of the frontal ‘ice ball’. New monitoring techniques using MRI are in development [23]. The current equipment is the third generation.

Currently, cryosurgery is recognised as a therapeutic alternative by the UAE and the AUA [24]. Numerous studies show good oncological results with reduced morbidity associated with the treatment.

In 2006, Bahn and collaborators presented results following focal cryotherapy in 31 low- and intermediate-risk patients. With a mean follow-up of 70 months, the biochemical recurrence-free survival rate was 93%; CaP recurrence in follow-up biopsies was 4%; erectile function was preserved in 90% of patients and urinary continence in 100%. No complications were observed [26].

In 2007, Lambert published a series of 25 treatments for low-risk patients using focal cryotherapy. With a mean follow-up of 28 months, the biochemical recurrence-free survival rate was 84%; CaP recurrence in follow-up biopsies was 12%; erectile function was preserved in 71% of patients and urinary continence in 100%. No complications were observed [27]. In the same year, Ellis presented 60 low-, intermediate-, and high-risk cases treated with cryotherapy. With a mean follow-up of 15 months, the biochemical recurrence-free survival rate was 80%; CaP recurrence in follow-up biopsies was 23%; erectile function was preserved in 71% of patients and urinary continence in 96%. No complications were observed. Patients with recurrence received repeat cryotherapy with good results [28].

In 2007–2008, Onik published a series of 55 cases. Patients were low, intermediate, and high risk with a mean follow-up of 4.5 years. At the end of the study, 94% were free of biochemical recurrence; recurrence in follow-up biopsies was 10%, predominantly in the high-risk group; erectile function was preserved in 90% of patients, and urinary continence in 100%. No complications were observed [29].

In 2010, Trusdale published the results of focal treatment in 77 low-, intermediate-, and high-risk patients. With a mean follow-up of 28 months, the biochemical recurrence-free survival rate was 73%; recurrence in follow-up biopsies was 13%; there was no difference in erectile function, and urinary continence was preserved in 100% of cases. No complications were observed [30].

### High-intensity focused ultrasound (HIFU)

HIFU consists of focused ultrasound waves emitted by a transducer that causes tissue damage through its thermal effects and cavitation [31]. Ultrasound waves pass through the tissue until they get to a specific point, with a reserve of insignificant energy on the journey and in neighbouring tissues; the absorption of waves by the tissue is transformed into heat. The heat damage is classified into three groups: the hyperthermia that can destroy cancer cells with temperatures between 41 and 19 °C in a period longer than 10 min; the coagulation that consists of tissue necrosis by the denaturing of proteins at temperatures above 60 °C for several minutes; and volatilisation that produces immediate necrosis of the tissue at temperatures above 100 °C (Figure 1). Cavitation is the interaction between the ultrasound and the water bubbles, which produce sudden collapses and energy dispersion by improving tissue ablation (1,27).

HIFU is administered under general or spinal anaesthetic, with the patient in the lateral recumbent position. Transrectal access is minimally invasive. A cooling system minimises the potential damage to the rectum and urethra [24]. At the moment there are two enabled systems: Ablatherm and Sonoblate 500.

Currently, HIFU is considered an experimental treatment by EUA and AUA [24]. Below is the scientific evidence so far: 

In 1995, Madersbacher proposed the use of HIFU for the treatment of localised CaP. The HIFU results had previously been evaluated in 29 patients who then underwent PR. The focal distances were checked, boundary of the lesion, interstitial thermometry, and collateral damage. Subsequently, the HIFU focal was checked in ten cases in the clinical stages T2a and T2b in an attempt to destroy the tumour prior to PR; in three cases, the lesion was totally destroyed and in seven it was partially destroyed. The quality of life was not assessed. There were no complications [31].

In 2008, Muto evaluated 29 low-, intermediate-, and high-risk patients. Patients with the disease in a single lobe received prostate focal therapy; the rest underwent total ablation. After 24 months of follow-up, the recurrence-free survival biochemistry in patients with low, intermediate, and high risk was 83%, 54%, and 0% respectively; the recurrence in the follow-up biopsy at six months was 11%, and at 12 months it was 23%. All patients were continent. Sexual potency was not evaluated. There were no complications [32].

In 2011, El Fegoun presented a series of 12 cases of low and intermediate risk treated with hemiablation by HIFU. He carried out a follow-up every ten years on average. Recurrence-free survival biochemistry at five and ten years is 90% and 38% respectively; the recurrence in the follow-up biopsy at 12 months was 8%; all patients were continent. Sexual potency was not evaluated. As complications, there were two urinary tract infections and a case of urinary retention [33].

Finally, Ahmed published two series. The first was in 2011, where he evaluated 20 low- and intermediate-risk patients over 12 months. In the protocol, he included the use of RM and transperineal biopsies with template mapping. The recurrence in the follow-up biopsy at six months was 11%; 95% of the patients were continent and 95% maintained potency; regarding complications, 65% presented with haematuria and 30% with dysuria [34]. The second series was published in 2012; 41 low- and intermediate-risk patients were assessed during a period of 12 months. The recurrence in the follow-up biopsy at six months was 23%, while only 8% had significant CaP; continence was maintained at 100% and potency in 89% of the cases. Regarding complications, 22% presented with dysuria, 34% with urinary detritus, 17% with ITU, and one case presented with urinary retention [25].

### Focal photodynamic therapy (PDT)

PDT is based on the interaction between a photosensitive agent, light, and the oxygen in the body tissue. It was initially used to treat skin lesions and has been a proven modality in breast cancer, central nervous system, lung, oesophagus, cervix, and bladder.

The photosensitive agent can be administered by mouth or intravenously; the new-generation products such as Padeliprofin (WST-11) accumulate preferentially in the malignant tissue in a stable and inactive manner [35, 36]. When light of a specific wavelength is administered, the photosensitive agent is activated. This produces a release of heat, an emission of photons, and the ‘triplet state’. This state produces cytotoxicity by the formation of free radicals and oxygen intermediates after oxido-reduction [37]. The free oxygen induces necrosis and indirect apoptosis through inflammation. The photosensitive agent is destroyed by the radicals, decreasing its effectiveness and fluorescence [38]; this allows the testing of the treatment’s effectiveness.

The light is administered by intraprostatic laser fibres guided through a trasperineal ultrasound, although initially carried out transurethrally [39]. A darkened room is required to prevent skin lesions.

It is currently an experimental technique, and it will require more evidence to define its role in CaP focal therapy. A description of the most relevant studies is as follows:

In 1990, Windahl applied haematoporphyrin derivative to the remaining prostate after transurethral resectioning in two patients. A PSA reduction was observed, and a secondary biopsy was negative [39].

In 2003, Zaak applied 5-ALA to six patients by a transurethral, a transperineal, and an open route. A PSA reduction between 20% and 70% was observed [36].

In 2006, Moore performed hemiablation with temoporfirina in six patients through a transperineal route. Areas of necrosis were observed in RM, and a reduction in PSA between 14% and 67% was also observed. All the patients presented with a recurrence in the follow-up biopsy. One patient presented with sepsis as a complication [40].

In 2010, Arumainayagan carried out hemiablation with WST-11 in 40 patients through the transperineal route. Areas of necrosis were observed by MRI. The complications were two cases of urinary retention. This does not evaluate the quality of life [41].

In 2011, Azzouzi carried out hemiablation with WST-11 in 85 patients through the transperineal route. Necrosis was observed by MRI in 87% of the treated lobe. Regarding complications, two patients had prostatitis, one haematuria, one orchitis, one optic neuropathy, and one urethral stenosis [42].

### Focal laser ablation (FLA)

FLA is the application of laser with conductive fibres through needles through the transperineal route into the prostate. The laser type most commonly used is neodymium–yttrium–aluminium–garnet (NdYAG) of 1.064-nm and 830-nm diodes.

The thermal effect of the laser produces an increase in temperature in the target tissue, causing volatilisation, clotting, and the consequent secondary inflammatory response.

The procedure can be monitored through RM and thermometry in critical areas such as the apex and the straight [43, 44].

FLA is still an experimental technique. We still do not have enough scientific evidence regarding oncological and functional results. The following are the most relevant studies:

In 2010, Linder and collaborators carried out PR in four patients for one week after having carried out FLA. They showed complete ablation of the target areas in RM and histopathological study [45]. This same group presented the results of its study in phase 1 in 12 patients, where 50% were free of the disease and 67% free of CaP in the treated lobe; they managed to maintain potency and continence [46].

## New therapies and approaches

### Transurethral resection of the prostate (TUR-P)

The TUR-P is a common procedure done primarily for the treatment of benign prostatic hyperplasia. However currently it is intended to give it new uses.

In 2012, Morita and colleagues presented the results of treatment with TUR-P of 79 patients with localised CaP. After 59 months of monitoring, the biochemical recurrence was 5.1%. Recurrence-free survival biochemistry was from 90% to 98% after two years. All patients were continent, and one presented with stenosis of the neck of the bladder [48]. There have not been any other studies since then.

### Thermal therapy with interstitial microwaves

Through tubes through the transperineal route micro-electromagnetic waves at frequencies from 300 to 2450 MHz are launched within the prostate tissue. These waves cause an increase in temperature, with the resulting tissue damage. The healthy prostate tissue, urethra, and rectum are protected through the use of cold-water systems.

This type of therapy has been tested regarding the recurrence of CaP after external radiotherapy (RT) [49]; at the moment, there is no evidence regarding its use for the treatment of focal CaP.

### Interstitial radio frequency (RITA)

The energy for the radio frequency is produced by a 50-W generator with a frequency of 460 kHz. It is administered through 15 needles with electrodes separated by an angle of 120°. Each electrode affects an area of 2 cm volume. The needles are inserted through the transperineal route and guided by ultrasound. RITA produces an increase in temperature until the central volatilisation in some cases and coagulation in the surrounding areas [50]. The urethra is preserved by cold irrigation systems.

In 1998, Zlotta showed that RITA therapy is feasible, safe, and reproducible for localised CaP [50].

In 2005, Shariat and colleagues presented 11 procedures of RITA, eight after recurrence after RT and three as a primary focal therapy. They demonstrated a decrease in the PSA greater than 50% in 90% of patients; the time of duplication of PSA after RITA was slower than that before the treatment (37 versus 14 months); the follow-up biopsy at 12 months was negative in 55% of the cases, and in 67%, it was negative in the treated areas. It had only minor complications (two haematuria, three dysurias). The quality of life was not assessed [51].

### Irreversible electroporation (IRE)

The IRE is a new method of non-thermal ablation that uses short electrical pulses to create irreversible pores in cell membranes.

In a preclinical study on six dogs, Onik showed the effectiveness of this therapy for the focal treatment of CaP [52]. It is important to emphasise that there is great potential to develop with this technology. There are important protocols in progress to test the direct application of IRE in the focal therapy of prostate cancer.

### Thermotherapy with nanoparticles

This is a new method of interstitial thermotherapy followed by the injection of electromagnetic nanoparticles.

In a study of ten patients with a recurrence of CaP after external radiation therapy (RT), its effectiveness has been demonstrated; as for disadvantages, it has discomfort for patients, which is generated by its strong magnetic fields and the irregular distribution of intra-tumour heat [53].

### External radiation therapy (RT), brachytherapy (BT), cyberknife, and proton therapy

Radiation is traditionally administered globally through external RT and BT, and now thanks to the technological development, it can be focussed and can vary the intensity of the dose [2].

In a systematic review of multiple studies by Anudh, it has been proved that this experimental technique of RT and focal BT has shown good preliminary results for the time being but it requires more study [47].

Proton therapy appears as another viable option for the focal therapy of CaP; however, its cost–benefit counts it out as the most viable option [54].

The cyberknife is a new technique that could offer oncological results similar to RT and BT with less morbidity. Controlled studies are needed to confirm these results [1].

## Cases of recurrence

Once the primary focal treatment is done, the later follow-up is performed in accordance with the guidelines of the EUA and AUA [24]. PSA and digital rectal examination must be performed at three, six, and 12 months during the first year; every six months for the next three years; and subsequently on an annual basis. The criteria for biochemical recurrence in focal therapy are not yet established; however, the trend is to take the current recommendations of the American Society for Therapeutic Radiology and Oncology for the RT, in which a PSA value per enzyme of 2 ng/ml from the nadir is considered positive [55]. Some authors set this threshold at 1 ng/ml [56].

The follow-up biopsy is recommended only in cases of recurrence biochemistry for the diagnosis of local recurrence in cases where it is going to influence a secondary treatment [24]. However, focal therapy, being an alternative experimental therapy, requires a biopsy by protocol, although there is no clear recommendation. Some studies have shown that, in the follow-up biopsy, cases of recurrence without elevation of the PSA [57, 58] can be detected and could support this approach. The moment at which the biopsy must be performed is not clearly defined; it is usually performed between six and 12 months post-focal treatment. The recommendation of the EUA in cases suspected of local recurrence after global therapy is carried out at 18 months [24].

**Figure 2.**
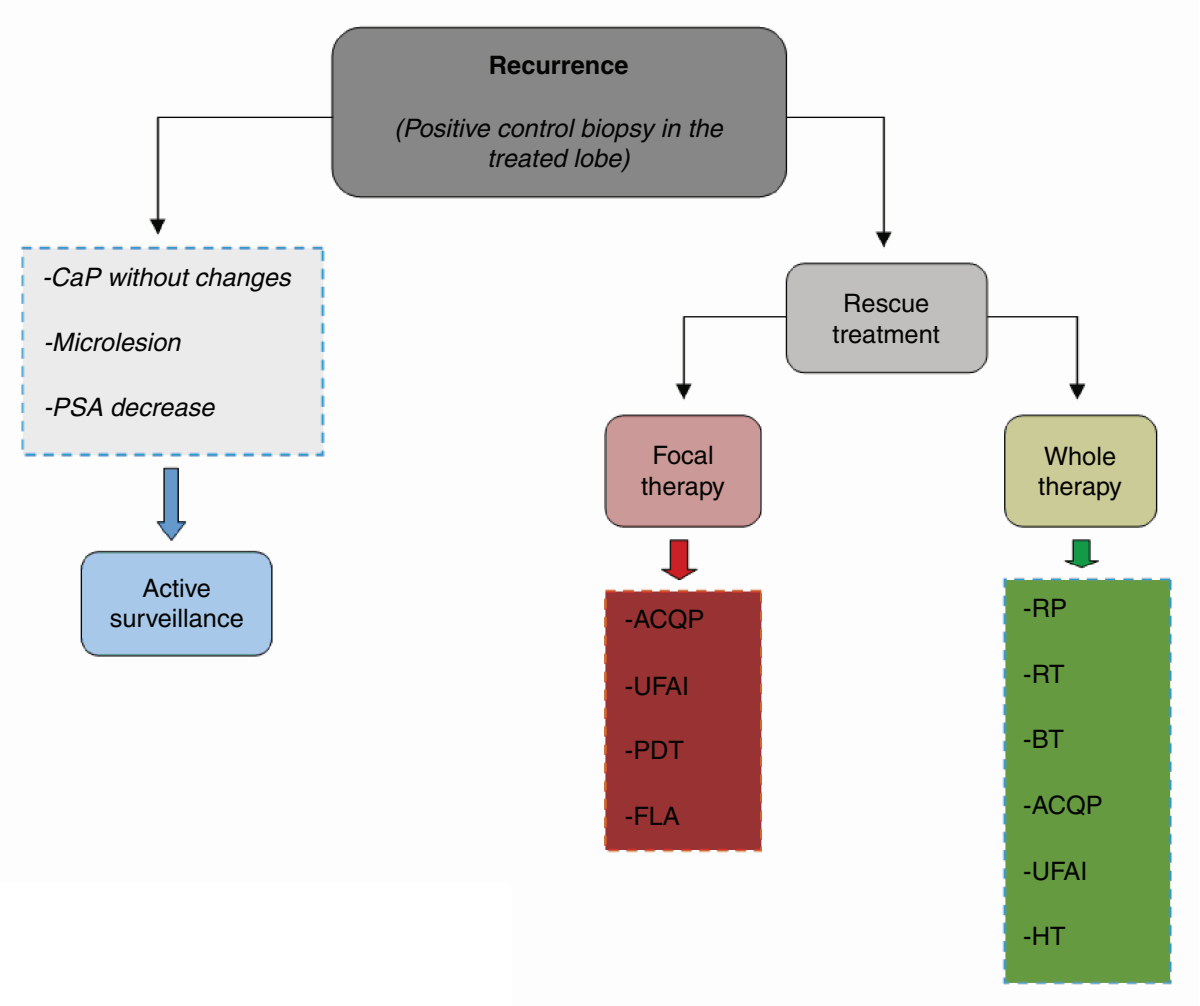
Treatment algorithm.

The three scenarios that can occur after the result of the control biopsy are (1) negative biopsy; (2) positive biopsy in the treated lobe; (3) positive biopsy in the untreated lobe. The genuine cases of recurrence in focal therapy are those with a positive biopsy in the treated prostate lobe (Figure 2).

Retreatment of cases of recurrence is determined by kinetics and PSA levels, and the histopathological result in the control biopsy. In the case of local recurrence without evidence of systemic disease, the most logical option of secondary treatment would be the use of the same approach and initial vigour. It is necessary to take into account the limitations of each focal therapy (Table 1) and be aware that a different secondary therapy can always be used. These recommendations still require scientific validation.

## Conclusion

Focal therapy in prostate cancer is emerging as a realistic alternative to radical treatment, because it could preserve sexual function and urinary continence; however, studies with greater scientific validity to check its effectiveness in oncology are needed.

## Conflicts of Interest

The authors have no conflicts of interest to declare.

## Figures and Tables

**Table 1.** Limitations of each focal therapy.

ACQP	UFAI	TDF	FLA	RT/BT
Voluminous Prostate	Voluminous Prostate			Voluminous Prostate
Anterior tumour	Anterior tumour	Anterior tumour	Anterior tumour	
Cost	Cost			
		Little experience	Little experience	
				Previous TUR-P
